# HACE1 Negatively Regulates Virus-Triggered Type I IFN Signaling by Impeding the Formation of the MAVS-TRAF3 Complex

**DOI:** 10.3390/v8050146

**Published:** 2016-05-21

**Authors:** He-Ting Mao, Yan Wang, Juan Cai, Jun-Ling Meng, Yu Zhou, Yu Pan, Xiao-Ping Qian, Yu Zhang, Jun Zhang

**Affiliations:** Key Laboratory of Medical Immunology (National Health and Family Planning Commission of China), Department of Immunology, School of Basic Medical Sciences, Peking University Health Science Center, Beijing 100191, China; maoheting@126.com (H.-T.M.); wangyan_tcell@hotmail.com (Y.W.); caijuan1919@163.com (J.C.); mengjunling46@sina.com (J.-L.M.); germany163@sina.com (Y.Z.); panyuzhaoning@163.com (Y.P.); qianxp1959@163.com (X.-P.Q.); zhangyu007@bjmu.edu.cn (Y.Z.)

**Keywords:** HACE1, TRAF3, MAVS, interferon, inflammation

## Abstract

During virus infection, the cascade signaling pathway that leads to the production of proinflammatory cytokines is controlled at multiple levels to avoid detrimental overreaction. HACE1 has been characterized as an important tumor suppressor. Here, we identified HACE1 as an important negative regulator of virus-triggered type I IFN signaling. Overexpression of HACE1 inhibited Sendai virus- or poly (I:C)-induced signaling and resulted in reduced IFNB1 production and enhanced virus replication. Knockdown of HACE1 expression exhibited the opposite effects. Ubiquitin E3 ligase activity of the dead mutant HACE1/C876A had a comparable inhibitory function as WT HACE1, suggesting that the suppressive function of HACE1 on virus-induced signaling is independent of its E3 ligase activity. Further study indicated that HACE1 acted downstream of MAVS and upstream of TBK1. Mechanistic studies showed that HACE1 exerts its inhibitory role on virus-induced signaling by disrupting the MAVS-TRAF3 complex. Therefore, we uncovered a novel function of HACE1 in innate immunity regulation.

## 1. Introduction

To initiate an effective antiviral response, RNA viruses are recognized by pattern recognition receptors (PRRs), such as Toll-like receptors (TLRs) and RIG-I-like receptors (RLRs), and then trigger multiple signaling pathways to promote the production of proinflammatory cytokines, including type I IFNs [1,2,3,4]. Aberrant overreaction may lead to proinflammatory diseases. Therefore, the intensity and duration of the signaling are finely modulated at multiple steps of the signaling cascades [5,6]. In recent years, we have had great interest in the identification of the essential regulators in this signaling pathway. This will provide potential therapeutic intervention and targets for infection, inflammation or autoimmune diseases in the future.

RLRs are cytosol sensors, which include RIG-I, melanoma differentiation factor 5 (MDA5) and laboratory of genetics and physiology 2 (LGP2) [7,8]. All three RLRs possess a DEXD-box RNA helicase domain for RNA binding [9]. Except LGP2, both RIG-I and MDA5 also contain a caspase recruitment domain (CARD) that is indispensable for downstream protein-protein interactions. Upon viral infection, the activated RIG-I undergoes self-dimerization and structural changes that permit the CARD domain of RIG-I to interact with the CARD domain of downstream essential adaptor protein MAVS (also known as IPS-1/Cardif/VISA) [10,11,12,13]. MAVS has a transmembrane domain (TM), that guides it to the outer mitochondrial membrane. Besides, MAVS contains three TRAF-interacting motifs (TIM), two included in the N-terminal proline-rich region (Pro), the other one located in the C-terminal region [11,13]. Upon RNA virus infection, the downstream tumor necrosis factor (TNF) receptor-associated factors (TRAFs) are recruited to MAVS, and the MAVS complex is formed [11,14]. This is a crucial step to initiate type I IFN signaling. TRAF2, 3, 5 and 6 are all MAVS binding partners through different TIM. The MAVS-TRAF3 complex provides the essential platform for downstream TBK1-dependent IRF3 or IRF7 activation. TRAF3 bridges the upstream MAVS and downstream kinase TBK1 and assembles the active MAVS-TRAF3-TBK1 signaling complex [14,15]. Therefore, the regulation on the MAVS-TRAF3 signalosome may be very important for the pathway.

HACE1 (HECT domain and ankyrin repeat-containing E3 ubiquitin protein ligase 1) is a HECT-type ubiquitin E3 ligase. The functions of HACE1 have not been fully understood. Until now, the identified ubiquitinated substrates of HACE1 include active Rac1 [16,17], optineurin (OPTN) [18] and Rab GTPases [19]. The catalytic cysteine (C876) of HACE1 is essential for its E3 ligase activity [16,20,21]. Mutation of C876 to serine or alanine will abolish its E3 ligase activity. HACE1 gene is located on chromosome 6q21, a prominent tumor-suppressor region [20,22]. The tumor suppressive function of HACE1 is also characterized. HACE1 is downregulated in multiple cancer types due to allelic loss or promoter methylation, such as Wilms’ tumor, gastric cancer, lymphoma, hepatocellular carcinoma, breast cancer, neuroblastoma, advanced colorectal cancer, *etc.* [23,24,25,26,27,28,29]. HACE1-deficient mice developed spontaneous, late-onset cancer [20]. Re-expression of HACE1 in human tumor cells directly abrogates *in vitro* and *in vivo* tumor growth, which is dependent on its E3 ligase activity. The mechanical analysis for its growth control shows that HACE1 modulates the expression level of cyclin D1, then reducing cell cycle progression [20]. Moreover, in breast cancer, HACE1 ubiquitinates and promotes the degradation of Rac1, then leading to impaired Rac signaling [29]. In contrast, HACE1 deficiency results in enhanced Rac1 signaling, contributing to breast cancer progression [29,30,31]. In lung cancer, HACE1 ubiquitinates OPTN and targets it for autophagic degradation. The HACE1-OPTN axis synergistically suppresses the growth and tumorigenicity of lung cancer cells [18]. Moreover, HACE1 is also involved in other biological processes or pathological conditions. For example, HACE1 mediates resistance to oxidative stress [32]. HACE1 regulates Golgi membrane fusion in cells [33]. It has protective roles in the pathology of neurodegenerative diseases, such as Huntington disease [32]. It also provides cardiac protection in response to hemodynamic stress [34]. However, the functions of HACE1 in immune responses are not investigated.

In recent years, ubiquitination has been reported as an important post-transcriptional modification to control the duration and intensity of antiviral immune responses [35]. Both HECT and RING domain E3 ubiquitin ligases are identified as essential regulators in this pathway. For example, RNF125 is reported to ubiquitinate and degrade MDA-5, RIG-I and MAVS [36]. The HECT domain containing ubiquitin ligase AIP4 can ubiquitinate and degrade MAVS in collaboration with PCBP2 [37]. Our group previously showed that Smurf2 promotes the ubiquitination and degradation of MAVS, as well [35]. In the search for unknown ubiquitin E3 ligases involved in antiviral signaling, some ubiquitin E3 ligases were used for the dual reporter luciferase assay. Then, HACE1 was suggested as a potential candidate in the regulation of this pathway.

In this study, we demonstrate for the first time that HACE1 contributes to negative regulation of the virus-induced type I IFN signaling via disrupting the MAVS-TRAF3 complex. HACE1 suppressed virus-induced type I IFN signaling independently of its ubiquitin E3 ligase activity. This study highlights the importance of HACE1 in the modulation of virus-induced type I IFN response.

## 2. Materials and Methods

### 2.1. Cells and Reagents

HEK293T and HEK293 cells were cultured with high-glucose DMEM (Life Technologies, New York, NY, USA) medium plus 10% heat-inactivated new-born bovine serum and supplemented with antibiotics (100 U/mL penicillin, 100 µg/mL streptomycin). Cells were grown at 37 °C in a humidified atmosphere with 5% CO_2_.

Mouse anti-Flag (M2) (Sigma-Aldrich, St. Louis, MO, USA), mouse anti-hemagglutinin (HA) (Merck Millipore, Darmstadt, Germany), anti-GAPDH (BioWorld, Atlanta, GA, USA), anti-HACE1 (Abcam, Cambridge, UK) and anti-GFP (Neobioscience, Shenzhen, China) were from the indicated manufacturers.

### 2.2. Plasmids

Mammalian expression plasmids for human HA-tagged HACE1 and Flag-tagged Rac1 were constructed by inserting the open reading frame of HACE1 or Rac1 into the N terminal HA or Flag-tagged pRK vector. The mammalian expression plasmid for HACE1/C876A was constructed by site-directed mutagenesis. All of these vectors were verified by sequencing. pcDNA3-Flag-TBK1 was a gift from Tom Maniatis. pEF-Bos-Flag-RIG-I was a gift from Takashi Fujita. pcDNA3-Flag-MAVS was a gift from Zhijian Chen. The pRL-TK-Renilla luciferase plasmid was from Promega (Madison, WI, USA). IFN-β and ISRE luciferase reporter plasmids were provided by Hong-Bing Shu.

### 2.3. RNA Interference

All small interfering RNAs (siRNAs) (Gene-Pharma, Shanghai, China) were transfected by PerMute (UcallM, Jiangsu, China) at 50 nM according to the manufacturers’ instructions. To determine the efficiency of protein knockdown, at 48 h post-transfection, cells were harvested, lysed and immunoblotted with rabbit anti-HACE1 Ab. The sequences of the individual siRNAs were as follows: nonspecific control, 5′-UUCUCCGAACGUGUCACGU-3′; HACE1 #1, 5′-UAUAGCGCUGAUGUCAACA-3′; HACE1 #2, 5′-GGUCUGUUUCUGAACUACU-3′ [20].

### 2.4. Luciferase Assays

The luciferase assay was performed as described [38]. Cells (1.1 × 10^5^) were seeded on 24-well plates and transfected the next day using VigoFect (Vigorous Biotechnology, Beijing, China) with 100 ng ISRE luciferase reporter, or IFN-β reporter and 1 ng pRL-SV40 plasmid, or with indicated plasmids. In the same experiment, when necessary, an empty control plasmid was added to ensure that each transfection received the same amount of total DNA. Then, 24 h after transfection, cells were infected with SeV at the multiplicity of infection (MOI) of 20 or transfected with poly (I:C) (InvivoGen, San Diego, CA, USA) using Lipofectamine 2000 (Invitrogen, Carlsbad, CA, USA) for 24 h, and luciferase activity was measured with the Dual-Luciferase reporter assay system (Promega) according to the manufacturer’s instructions. Firefly luciferase activity was normalized based on Renilla luciferase activity. All reporter assays were performed in duplicate and repeated at least three times. The representative results are shown in each figure.

### 2.5. RT-PCR and Real-Time PCR

Total RNA was isolated using TRIzol reagent (Life Technologies). cDNA was synthesized using a reverse transcription system (Promega) according to the manufacturer’s instructions. Quantitative real-time polymerase chain reaction (PCR) was carried out with the Power SYBR Green PCR master mix (Bio-Rad, Berkeley, CA, USA). Each reaction was in duplicate. The amounts of *hIFNB1* were amplified using the following primers: *IFNB1-F*: 5′-ATTGCCTCAAGGACAGGATG-3′ and *IFNB1-R*: 5′-GGCCTTCAGGTAATGCAGAA-3′; for monitoring of VSV (Vesicular Stomatitis Virus) infection, the cells were infected by VSV-GFP virus at MOI of 10. Then, the VSV genome was quantified by real-time PCR using the following primers: *VSV-F:* 5′-ACGGCGTACTTCCAGATGG-3′; *VSV-R:* 5′-CTCGGTTCAAGATCCAGGT-3′.

### 2.6. Co-Immunoprecipitation and Immunoblot Analysis

HEK293T cells were transfected with indicated plasmids for 24 h. Then, the cells were lysed in lysis buffer containing a proteinase inhibitor mixture (Roche, Indianapolis, IN, USA) and PMSF. Cell lysates were incubated with 1 µg/mL anti-HA Ab or anti-Flag or control Ig (IgG) and protein A-Sepharose (GE Healthcare, GE Healthcare, Calbiochem, Sweden) and resolved by SDS-PAGE. The blot was then probed with anti-Flag or anti-HA Ab. IRDye 700-conjugated anti-IgG or HRP-conjugated anti-IgG was used as a secondary Ab, and proteins were identified using the Odyssey imaging system or detected by the ECL assay.

### 2.7. Statistical Analysis

Statistical analysis was carried out with SPSS 13.0. All data are shown as the mean ± SD. The mean values from each group were compared by Student’s *t*-test. In all tests, *p*-values of less than 0.05 were considered statistically significant.

## 3. Results

### 3.1. HACE1 Negatively Regulates Virus-Induced Type I IFN Signaling

**Figure 1:**
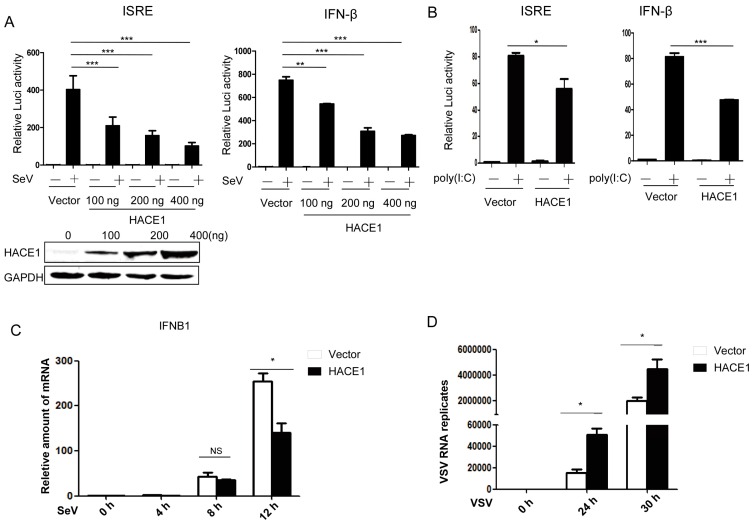
HACE1 negatively regulates virus-induced type I IFN signaling. (**A**) Overexpression of HACE1 inhibited SeV-induced ISRE and IFN-β promoter activation in a dose-dependent manner; (**B**) HACE1 inhibited ISRE and IFN-β promoter activation by transfected poly (I:C) in HEK293T cells; (**C**) overexpression of HACE1 inhibited SeV-induced transcription of *IFNB1*. HEK293T cells were seeded on 24-well plates and transfected the next day with mock control or HACE1-expressing vector. Twenty-four hours later, cells were left uninfected or infected with SeV. Cells were harvested at the indicated time point; (**D**) Overexpression of HACE1 promoted VSV replication. HEK293T cells were seeded on 24-well plates and transfected the next day with mock control or HACE1-expressing vector. Twelve hours later, cells were infected with VSV-GFP. Cells were harvested at the indicated time point. For (**A**,**B**), all data are representative of at least three independent experiments. For (**C**,**D**), all data are representative of at least two independent experiments. * *p* < 0.05; ** *p* < 0.01; *** *p* < 0.001.

By a small-scale screening of unknown ubiquitin E3 ligases in the regulation of virus-induced type I IFN signaling by the dual-luciferase reporter, we identified HACE1 as a potential negative regulator in this pathway. Then, we tried to systematically investigate whether HACE1 is indeed involved in the regulation of virus-induced IFN signaling. As shown in Figure 1A, overexpression of HACE1 inhibited SeV-induced activation of both ISRE (an interferon stimulated response element) and IFN-β promoter in a dose-dependent manner in HEK293T cells. In addition, activation of the ISRE promoter primed with the synthetic RNA duplex poly (I:C) was also inhibited by overexpression of HACE1 (Figure 1B). To further support these results, the amount of *IFNB1* was measured at various time points by reverse transcription (RT)-PCR during the twelve hours of infection by Sendai virus. HACE1 suppressed SeV-induced transcription of endogenous *IFNB1* gene (Figure 1C). VSV is another representative RNA virus for RIG-I signaling studies. It is easy to detect the virus replication. Consistent with the suppressive function of HACE1 on virus-induced signaling, the replication of VSV was enhanced when HACE1 was overexpressed (Figure 1D). These data together suggested that HACE1 negatively regulates virus-induced type I IFN signaling.

### 3.2. Knockdown of HACE1 Augments Virus-Induced Type I IFN Signaling

**Figure 2:**
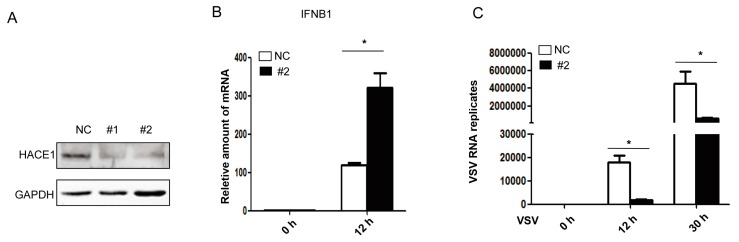
HACE1 knockdown augments anti-viral signaling. (**A**) HEK293 cells were transfected with control or siRNA oligos against HACE1; then 36 h later, the knockdown efficiency was monitored by Western blot; (**B**) Knockdown of HACE1 augmented SeV-induced *IFNB1* production; (**C**) knockdown of HACE1 inhibited VSV replication. HEK293 cells were seeded on 12-well plates and transfected the next day with control or HACE1 #2 siRNA at 50 nM. Thirty six hours later, cells were infected with VSV-GFP. Cells were harvested at the indicated time point. Data are representative of at least two independent experiments. * *p* < 0.05.

Next, to investigate the functions of endogenous HACE1 on virus-induced type I IFN signaling, we knocked down the expression of HACE1 in HEK293 cells. Two siRNA oligos against HACE1 were used, and the knockdown efficiency was monitored. As shown in Figure 2A, both #1 and #2 siRNA oligos can remarkably reduce the expression of endogenous HACE1 in HEK293 cells. Compared to control cells, knockdown of HACE1 expression augmented SeV-induced *IFNB1* gene transcription (Figure 2B) and inhibited VSV replication (Figure 2C). Thus, HACE1 is a negative regulator in virus-induced type I IFN signaling.

### 3.3. The Suppressive Function of HACE1 Is Independent of Its E3 Ligase Activity

**Figure 3:**
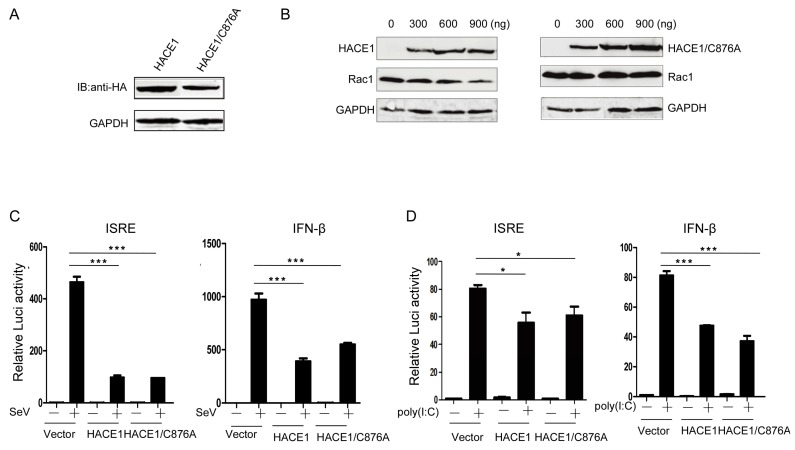
HACE1 inhibited antiviral signaling independently of its ubiquitin E3 ligase activity. (**A**) The expression of sequencing verified that HACE1/C876A was monitored by Western Blot. HACE1/C876A expression vector; (**B**) HACE1/C876A lost the ability to promote the degradation of Rac1. HEK293T cells were coexpressed with Rac1 and increasing amounts of HA-tagged WT HACE1 or HACE1/C876A. Twenty-four hours after transfection, the cells were harvested and detected by Western blot; (**C**) HACE1/C876A still has the ability to inhibit SeV-induced ISRE or IFN-β activation. HEK293T cells were seeded on 24-well plates and were transfected the next day with mock control, HACE1 or HACE1/C876A expressing vector, together with ISRE or IFN-β reporter vector. Twenty-four hours later, cells were infected with SeV or left uninfected for 24 h before luciferase assays were performed; (**D**) HACE1/C876A inhibited ISRE and IFN-β promoter activation by transfected poly (I:C) in HEK293T cells. Data are representative of at least three independent experiments. * *p* < 0.05; *** *p* < 0.001.

HACE1 has been documented to act as a ubiquitin E3 ligase. So far, only a few ubiquitinated substrates have been identified, including Rac1, Optineurin (OPTN) and Rab GTPases [16,18,19]. The catalytic cysteine (C876) is indispensable for its E3 ubiquitin ligase activity [16,20,21]. Then, we tried to determine whether the E3 ligase activity of HACE1 is required for the inhibition of virus-induced type I IFN signaling. It is well-known that the catalytic cysteine (C876) of HACE1 is indispensable for its E3 ligase activity [20]. Therefore, we constructed a mutant HACE1 in which the amino acid cysteine 876 was mutated into alanine (Figure 3A). Consistent with the previous report, WT HACE1 can promote the degradation of Rac1, whereas the HACE1/C876A mutant lost the ability to degrade Rac1, indicating the lost E3 ligase activity of HACE1/C876A (Figure 3B). Reporter assays showed that SeV-induced or poly (I:C)-induced activation of ISRE and IFN-β promoter activities were inhibited by HACE1/C876A to a similar degree as WT HACE1 (Figure 3C,D). These data indicate that HACE1 inhibits virus-induced type I IFN induction independently of its E3 ligase activity.

### 3.4. HACE1 Negatively Regulates Virus-Triggered Signaling Downstream of MAVS and Upstream of TBK1

**Figure 4:**
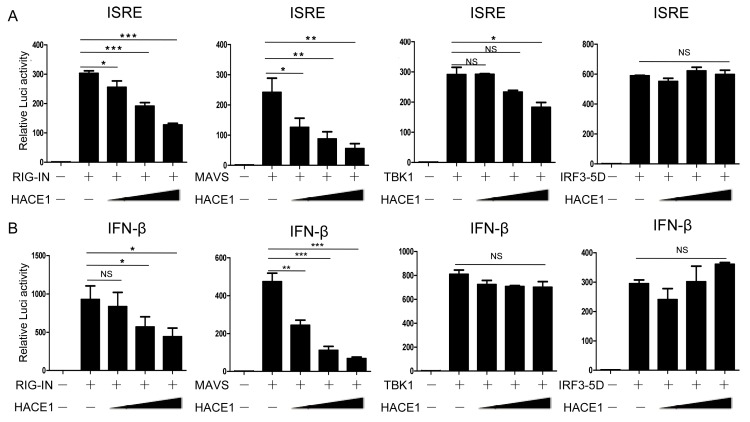
HACE1 targets downstream of MAVS and upstream of TBK1. (**A**) Effects of HACE1 on ISRE activation by various signaling components in a dose-dependent manner; (**B**) effects of HACE1 on IFN-β activation by various signaling components in a dose-dependent manner. HEK293T cells were seeded on 24-well plates and transfected the next day with indicated signaling molecules, IFN-β or ISRE luciferase reporter and pRL-SV40 plasmid and increasing doses of HACE1 for 24 h. The luciferase activities were quantified by normalizing with Renilla luciferase activities. Data are representative of at least three independent experiments. * *p* < 0.05; ** *p* < 0.01; *** *p* < 0.001.

Upon viral infection, recognition of viral RNA by RIG-I induced a downstream signaling cascade, including MAVS, TBK1, IRF3 [39]. We next sought to determine a step within the signaling pathway that HACE1 targets. As shown in Figure 4, ISRE and IFN-β promoter activity were activated by transfection of an active form of RIG-I (RIG-IN), MAVS, TBK1 and an activated form of IRF3 (IRF3/5D), respectively. Co-expression of HACE1 inhibited RIG-IN, MAVS-induced ISRE or IFN-β reporter activation (Figure 4A,B). On the other hand, it had no apparent effects on TBK1 or IRF3-5D-induced ISRE or IFN-β reporter activation. These data suggested that HACE1 acted downstream of MAVS and upstream of TBK1 in the virus-induced signaling.

### 3.5. HACE1 Suppresses Virus-Induced Signaling by Disrupting the MAVS-TRAF3 Complex

**Figure 5:**
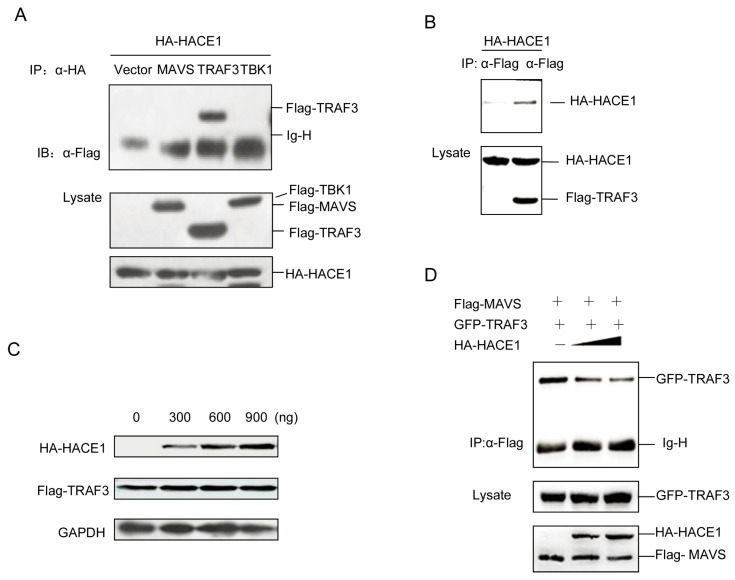
HACE1 impedes the formation of the MAVS-TRAF3 complex. (**A**,**B**) HACE1 interacted with TRAF3 in a mammalian overexpression system. HEK293T cells were transfected with the mock control, Flag-tagged MAVS, Flag-tagged TRAF3 or Flag-tagged TBK1 and HA-tagged HACE1. Co-immunoprecipitation was performed with indicated antibodies, and then, the membrane was blotted with anti-Flag or anti-HA Ab; (**C**) HACE1 did not promote the degradation of TRAF3. HEK293T cells were coexpressed with Flag-tagged TRAF3 and increasing amounts of HA-tagged HACE1. Twenty-four hours after transfection, the cells were harvested and detected by Western blot. (**D**) HACE1 disrupts the MAVS-TRAF3 complex. HEK293 cells were transfected with Flag-MAVS and GFP-TRAF3 with an increasing amount of HA-HACE1. Flag-MAVS was immunoprecipitated, and the membrane was immunoblotted with anti-GFP antibody. Data are representative of at least three independent experiments.

As suggested by Figure 4, HACE1 modulated the virus-induced signaling downstream of MAVS and upstream of TBK1. Then, we tried to investigate the exact mechanisms underlying the suppressive function of HACE1. We coexpressed HACE1 with MAVS, TRAF3 and TBK1. Unexpectedly, HACE1 cannot interact with either MAVS or TBK1. It interacted with TRAF3 (Figure 5A,B). HACE1 is a HECT ubiquitin E3 ligase. Then, we tested whether HACE1 can promote the degradation of TRAF3. As shown in Figure 5C, HACE1 cannot degrade TRAF3. This is consistent with the results above (Figure 3C,D) that the HACE1/C876A mutant has a comparable inhibitory function to WT HACE1 on virus-induced signaling.

It has been reported that the MAVS and TRAF3 complex is essential for virus-induced signaling [14,15]. Then, we detected the impact of HACE1 expression on the MAVS-TRAF3 complex. With the increase of the expression level of HACE1, the formation of the complex of MAVS-TRAF3 was severely impaired (Figure 5D). These data indicate that HACE1 may negatively regulate the virus-induced signaling by disrupting the MAVS-TRAF3 complex.

## 4. Discussion

In the present study, we provide evidence for the first time that HACE1 is an important negative regulator of virus-induced type I IFN signaling. Additionally, this suppressive function is E3 ligase activity independent. HACE1 plays its suppressive role downstream of MAVS and upstream of TBK1. Co-immunoprecipitation assays showed that HACE1 did not interact with MAVS or TBK1. Unexpectedly, it binds TRAF3, which interacts with MAVS and forms a platform for RNA virus signaling. HACE1 does not promote the degradation of TRAF3. This is consistent with the data that HACE1 negatively regulates the virus-induced signaling independent of the E3 ligase activity. Further studies shown that HACE1 can disrupt the MAVS-TRAF3 complex. This provides a mechanistic explanation for the suppressive function of HACE1 on virus-induced innate immune response.

HACE1 is a HECT-type E3 ligase. The most studied function of HACE1 is its involvement in tumor development. This function is E3 ligase dependent [40]. HACE1 can also function in an E3 ligase independent manner. For example, HACE1 can repress the transcriptional activity of RARα1 and RARβ3. Mutation of the putative catalytic cysteine (C876) does not alter the repressive effect of HACE1 on the transcriptional activity of RARβ3 [21]. HACE1 can mediate p62-dependent selective autophagic turnover of ubiquitinated proteins. This process is achieved by protein-protein interaction through its ankyrin repeat domain and is independent of its E3 ligase activity [34]. Under stress conditions, HACE1 cleared the ubiquitinated proteins in an E3 ligase activity independent manner. Here, we demonstrated that HACE1 suppressed virus-induced signaling independently of its E3 ligase activity, suggesting that HACE1 exerts diverse biological functions by different mechanisms.

Several viral or cellular negative regulators may hijack TRAF3 or the TRAF3 complex to mediate immune evasion. Herpes simplex virus 1 ubiquitin-specific protease UL36 deubiquitinates TRAF3 [41]. A few SARS coronavirus proteins are identified as viral negative regulators, which target the TRAF3 signalosome [42]. SARS coronavirus papain-like protease binds and disrupts the STING-TRAF3-TBK1 complex [43]. SARS coronavirus M protein or open-reading frame-9b impedes the formation of the TRAF3/TANK/TBK1/IKKε complex or the MAVS/TRAF3/TRAF6 complex, respectively [44]. Besides, some studies reported endogenous physiological negative regulators that target TRAF3 or the MAVS-TRAF3 complex. The linear ubiquitin assembly complex (LUBAC) downregulates virus-mediated IFN induction by targeting NEMO for linear ubiquitination. Then, linear ubiquitinated NEMO is associated with TRAF3 and disrupts the MAVS-TRAF3 complex, which inhibits IFN activation [45]. MIP-T3, a ciliary protein, is also a TRAF3 binding protein, which acts as a cellular inhibitor in virus-induced IFN production. MIP-T3 impedes the formation of multiple TRAF3 signaling complex, such as the MAVS-TRAF3 complex, the TRAF3-TBK1 or TRAF3-IKKε complex [46]. The ubiquitin E3 ligase Triad3A targets TRAF3 for degradation to negatively regulate the RIG-I signaling [47]. Here, we elucidate a novel role of HACE1 in virus-induced signaling, which also targets the central MAVS-TRAF3 complex.

The interaction of proteins with TRAF family members were mediated by the TRAF interaction motif. We also analyzed the structure of HACE1 and found that there is a potential TIM between amino acid 357 to amino acid 365 (FKPLELLWH); we mutated central amino acid PLE to AAA, and then performed the luciferase assays. The results showed that this mutant lost the ability to suppress virus-induced signaling, indicating that the suppressive function of HACE1 is dependent on the complete TRAF interaction motif. Further studies will focus on the link of the spontaneous mutation of HACE1 with inflammation or tumor development, which will be intriguing.

## 5. Conclusions

In this study, we identified a novel function of HACE1 on virus-induced type I IFN signaling, which targets the MAVS-TRAF3 complex and impedes the assembly of the MAVS-TRAF3 complex.
